# Association between endotoxemia and histological features of nonalcoholic fatty liver disease

**DOI:** 10.3748/wjg.v23.i4.712

**Published:** 2017-01-28

**Authors:** Hiroyuki Kitabatake, Naoki Tanaka, Naoyuki Fujimori, Michiharu Komatsu, Ayaka Okubo, Kyogo Kakegawa, Takefumi Kimura, Ayumi Sugiura, Tomoo Yamazaki, Soichiro Shibata, Yuki Ichikawa, Satoru Joshita, Takeji Umemura, Akihiro Matsumoto, Masayoshi Koinuma, Kenji Sano, Toshifumi Aoyama, Eiji Tanaka

**Affiliations:** Hiroyuki Kitabatake, Naoki Tanaka, Toshifumi Aoyama, Department of Metabolic Regulation, Shinshu University Graduate School of Medicine, Matsumoto 390-8621, Japan; Hiroyuki Kitabatake, Naoyuki Fujimori, Michiharu Komatsu, Ayaka Okubo, Kyogo Kakegawa, Takefumi Kimura, Ayumi Sugiura, Tomoo Yamazaki, Soichiro Shibata, Yuki Ichikawa, Satoru Joshita, Takeji Umemura, Akihiro Matsumoto, Eiji Tanaka, Department of Internal Medicine, Division of Gastroenterology, Shinshu University School of Medicine, Matsumoto 390-8621, Japan; Masayoshi Koinuma, Center for Clinical Research, Shinshu University Hospital, Matsumoto 390-8621, Japan; Masayoshi Koinuma, Faculity of Pharmaceutical Sciences, Teikyo Heisei University, Tokyo 164-8530, Japan; Kenji Sano, Department of Laboratory Medicine, Shinshu University Hospital, Matsumoto 390-8621, Japan

**Keywords:** Nonalcoholic steatohepatitis, Endotoxemia, Lipopolysaccharide-binding protein, EndoCab IgG, Fibrosis, Steatosis

## Abstract

**AIM:**

To assess whether surrogate biomarkers of endotoxemia were correlated with the histological features of nonalcoholic fatty liver disease (NAFLD).

**METHODS:**

One hundred twenty-six NAFLD patients who had undergone percutaneous liver biopsy were enrolled. Serum lipopolysaccharide (LPS)-binding protein (LBP) and anti-endotoxin core immunoglobulin G (EndoCab IgG) antibody concentrations at the time of liver biopsy were measured using the enzyme-linked immunosorbent assays to examine for relationships between biomarker levels and histological scores.

**RESULTS:**

Serum LBP concentration was significantly increased in nonalcoholic steatohepatitis (NASH) patients as compared with nonalcoholic fatty liver (NAFL) subjects and was correlated with steatosis (*r* = 0.38, *P* < 0.0001) and ballooning scores (*r* = 0.23, *P* = 0.01), but not with the severity of lobular inflammation or fibrosis. Multivariate linear regression analysis revealed that LBP was associated with steatosis score and circulating C-reactive protein, aspartate aminotransferase, and fibrinogen levels. Serum EndoCab IgG concentration was comparable between NASH and NAFL patients. No meaningful correlations were detected between EndoCab IgG and histological findings.

**CONCLUSION:**

LBP/EndoCab IgG were not correlated with lobular inflammation or fibrosis. More accurate LPS biomarkers are required to stringently assess the contribution of endotoxemia to conventional NASH.

**Core tip:** This is the first study simultaneously measuring two surrogate endotoxemia markers, lipopolysaccharide-binding protein (LBP) and EndoCab IgG, in biopsy-proven nonalcoholic fatty liver disease (NAFLD) patients in order to assess for relationships with the histological features of NAFLD. Serum LBP/EndoCab IgG were not correlated with lobular inflammation or fibrosis. It remains elusive whether portal endotoxemia promotes hepatitis/fibrosis in human conventional NAFLD/nonalcoholic steatohepatitis.

## INTRODUCTION

The prevalence of nonalcoholic fatty liver disease (NAFLD) is increasing worldwide. NAFLD includes a wide spectrum of disorders, ranging from nonalcoholic fatty liver (NAFL) to nonalcoholic steatohepatitis (NASH) and resultant liver cirrhosis and hepatocellular carcinoma[1-4]. NASH is characterized by the presence of hepatocyte ballooning, lobular inflammation and/or various degree of fibrosis in addition to macrovesicular steatosis[1-3]. Although several pathogenic factors, such as lipotoxicity, endoplasmic reticulum stress, iron accumulation, and inflammatory signaling, reportedly contribute to the progression from steatosis to steatohepatitis/steatofibrosis, the mechanism of NASH development has not been fully clarified.

Recent murine studies have demonstrated a key role of endotoxin/lipopolysaccharide (LPS) in the onset of NASH[5,6]. For example, repeated LPS injection into *ob/ob* mice led to steatohepatitis[7], while mice lacking the gene encoding Toll-like receptor (TLR) 4, a central molecule in LPS-mediated signaling, were resistant to NASH development[8]. It is generally accepted that Kupffer cells are activated when the gut mucosa becomes inflamed and fragile or when gut bacteria overgrow and LPS subsequently flows into the portal vein. However, it remains unclear whether the gut barrier is disrupted and portal LPS levels are elevated in typical obesity-, metabolic syndrome-related NAFLD patients without accompanying active inflammatory bowel disease[9,10] or a history of gastrointestinal surgery, such as pancreaticoduodenectomy and blind loop construction[11,12].

Another issue requiring attention when considering the contribution of endotoxin/LPS to human NASH is the lack of appropriate systems to determine portal LPS concentration. Since portal LPS is rapidly eliminated in the liver, systemic LPS levels often do not mirror those in the portal vein, and the half-life of circulating LPS is as short as 2 h[13]. These shortcomings obscure the evaluation of endotoxin/LPS contribution to NASH.

Circulating LPS-binding protein (LBP) and anti-endotoxin core immunoglobulin G (EndoCab IgG) antibody are commercially available surrogate markers of endotoxemia[10,14]. LBP is a soluble acute-phase protein that binds to bacterial endotoxin. When the liver senses bacterial endotoxin, LBP is rapidly synthesized by hepatocytes to neutralize the toxin and then secreted into the circulation. The EndoCab IgG assay measures endotoxin core antibodies to reflect the immune response against persistent endotoxin exposure. Since both of these biomarkers are more stable than LPS, they represent possible indicators of endotoxemia.

In the current study, serum levels of LBP and EndoCab IgG were measured in biopsy-proven NAFLD patients and their correlation with the histological severity of NAFLD was assessed to clarify associations between endotoxemia and NASH development.

## MATERIALS AND METHODS

### Patients

This study was approved by the Committee for Medical Ethics of Shinshu University School of Medicine (Approval number: 2802) and conducted in accordance with the 1983 revision of the Helsinki declaration of 1975. Informed written consent was obtained from all patients. One hundred twenty-six NAFLD patients who were admitted to Shinshu University Hospital between February 2009 and April 2015 for percutaneous liver biopsy were enrolled. NAFLD had been suspected based on the following criteria: (1) the presence of hepatorenal contrast and increased hepatic echogenicity on abdominal ultrasonography; (2) ethanol consumption of < 20 g/d; and (3) the absence of other causes of liver dysfunction, such as viral hepatitis, drug-induced liver injury, autoimmune liver diseases, primary sclerosing cholangitis, Wilson’s disease, hereditary hemochromatosis, and citrin deficiency[15-18]. The diagnosis of NAFLD/NASH was confirmed based on the histological findings of biopsied specimens.

Body weight and height were measured before liver biopsy performed in a fasting state. The presence of obesity was defined as a body mass index of ≥ 25 kg/m^2^ according to criteria released by the Japan Society for the Study of Obesity[19]. Medical information was also recorded, and the presence of hypertension, hyperlipidemia, and diabetes was evaluated as described previously[20,21]. Blood samples were obtained on the day of the liver biopsy in a fasting state and routine examinations, such as complete blood counts, coagulation, and blood chemistry that included serum aspartate aminotransferase (AST) and alanine aminotransferase (ALT), were carried out using standard laboratory methods. The remaining sera samples were immediately frozen and kept at -80 °C until further use.

### Histopathological analysis

Biopsy specimens were obtained from liver segment 5 or 8 using a 14-gauge needle as described previously and immediately fixed in 10% neutral formalin. Sections were cut at a 4-μm thickness and stained by means of the hematoxylin and eosin and Azan-Mallory methods. The histological activity of NAFLD was assessed by an independent expert pathologist (KS) in a blinded manner for the degrees of steatosis, lobular inflammation, ballooning, and fibrosis according to the system proposed by Kleiner et al[22], as steatosis grade 1: 5%-33% of hepatocytes affected, grade 2: 33%-66% of hepatocytes affected, and grade 3: > 66% of hepatocytes affected; lobular inflammation grade 0: no inflammatory foci, grade 1: < 2 foci per 200 × ﬁeld, grade 2: 2-4 foci per 200 × ﬁeld, and grade 3: > 4 foci per 200 × ﬁeld; ballooning grade 0: no ballooned hepatocytes, grade 1: a few ballooned hepatocytes, and grade 2: many/prominent ballooned hepatocytes; and fibrosis stage (F) 0: no fibrosis, F1: perisinusoidal, perivenular, or portal/periportal fibrosis, F2: perisinusoidal and portal/periportal fibrosis, F3: bridging fibrosis, and F4: cirrhosis. NASH was defined as the presence of macrovesicular steatosis (≥ 5% of hepatocytes affected) and hepatocyte ballooning with or without lobular inflammation and fibrosis. NAFLD patients having macrovesicular steatosis without ballooning were diagnosed as having NAFL. The NAFLD activity score (NAS) was calculated as the unweighted sum of the scores for steatosis (1-3), lobular inflammation (0-3), and ballooning (0-2), ranging from 1 to 8.

### Measurement of serum LBP and EndoCab IgG levels

Frozen serum samples obtained at the time of liver biopsy were diluted in 1000- and 200-fold with dilution buffer, and serum LBP and EndoCab IgG concentrations were measured in duplicates using the LBP ELISA kit (HK315-01, Hycult Biotech, Uden, the Netherlands) and the ENDOCAB ELISA kit (HK504, Hycult Biotech), respectively, according to the manufacturer’s instructions.

### Measurement of serum cytokeratin 18 fragment levels

Serum concentrations of caspase-cleaved cytokeratin 18 (CK18) fragments were measured using the M30 Apoptosense^®^ ELISA kit (VLVbio AB, Nacka, Sweden) as described previously[15].

### Statistical analysis

Data are expressed as number (percentage) or median (25^th^, 75^th^ percentiles). Comparisons between the groups were carried out using the Mann-Whitney *U* test, χ^2^ test, or one-way ANOVA with Bonferroni’s correction. Spearman’s test was adopted to examine for correlations among LBP, EndoCab IgG, and biochemical/histological data. Multivariate linear regression analysis was conducted to search for independent predictors of LBP. Statistical analyses were performed using StatFlex Ver6.0 software (Artech Co., Ltd., Osaka, Japan). A *P* value of < 0.05 was considered to be statistically significant.

## RESULTS

### Serum LBP/EndoCab IgG levels in NAFLD patients

**Figure 1:**
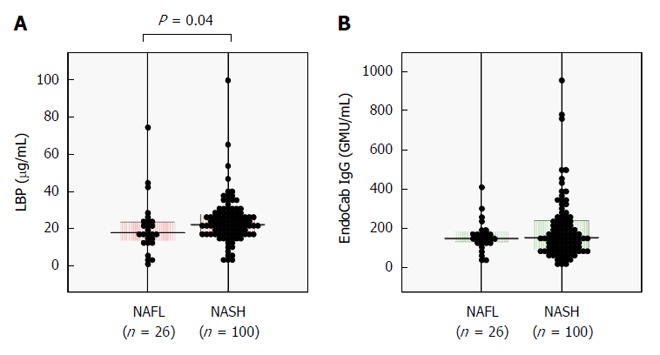
Comparisons of serum lipopolysaccharide-binding protein (A) and EndoCab IgG (B) between 26 nonalcoholic fatty liver and 100 nonalcoholic steatohepatitis patients. The clinicopathological features of the patients are shown in Table 1. Bars represent median values. *P* values were calculated using the Mann-Whitney *U* test. NAFL: Nonalcoholic fatty liver; NASH: Nonalcoholic steatohepatitis.

The clinicopathological features of the 126 NAFLD patients are summarized in Table 1. One-hundred patients (79%) were diagnosed as having NASH and 29 (23%) had advanced fibrosis of stage 3 or 4. Serum LBP concentration was significantly increased in NASH patients as compared with NAFL patients (Figure 1A), whereas serum EndoCab IgG was comparable between the groups (Figure 1B).

**Table 1:** Clinicopathological features of nonalcoholic fatty liver disease patients *n* (%)

**Parameter**	**All (*n* = 126)**	**NAFL (*n* = 26)**	**NASH (*n* = 100)**	***P* value**
Clinical findings				
Age (yr)	56 (44-65)	52 (42-58)	58 (45-65)	0.17
Male	54 (43)	14 (54)	40 (40)	0.20
BMI ≥ 25 kg/m^2^	76 (60)	12 (46)	64 (64)	0.10
Diabetes	43 (34)	5 (19)	38 (38)	0.07
Hypertension	49 (39)	8 (31)	41 (41)	0.34
Hyperlipidemia	82 (65)	19 (73)	63 (63)	0.34
BMI (kg/m^2^)	25.8 (23.6-29.4)	24.6 (22.6-28.5)	26.4 (23.9-29.5)	< 0.05
Ethanol (g/d)	0 (0-5)	0 (0-2.8)	0 (0-5)	0.26
Leukocytes (× 10^3^/μL)	5.57 (4.73-6.82)	5.07 (4.76-5.75)	5.72 (4.72-6.86)	0.24
Erythrocytes (× 10^4^/μL)	490 (453-515)	490 (474-512)	489 (452-515)	0.36
Hemoglobin (g/dL)	14.9 (14.0-15.8)	15.1 (14.6-15.9)	14.8 (13.9-15.8)	0.14
Hematocrit (%)	44.2 (41.6-46.6)	45.3 (43.3-46.9)	43.9 (41.5-46.6)	0.31
MCV (fl)	91.2 (88.5-93.7)	91.5 (89.1-93.0)	91.1 (88.4-94.1)	0.81
MCH (pg)	30.7 (29.6-31.7)	31 (30.2-31.6)	30.5 (29.6-31.7)	0.42
MCHC (%)	33.6 (33.0-34.3)	33.9 (33.5-34.4)	33.4 (32.9-34.2)	0.08
Platelets (× 10^4^/μL)	23.8 (17.9-26.8)	23.3 (19.1-26.7)	23.8 (17.8-26.9)	0.88
PT-INR	1.00 (0.98-1.05)	1.00 (0.98-1.03)	1.01 (0.97-1.05)	0.40
APTT (s)	28.6 (26.6-31.2)	28.6 (27.5-30.1)	28.6 (26.3-31.3)	0.84
FIBG (mg/dL)	287 (243-314)	284 (247-321)	287 (242-313)	0.73
Total protein (g/dL)	7.6 (7.3-7.9)	7.6 (7.3-7.9)	7.6 (7.3-7.8)	0.97
Albumin (g/dL)	4.5 (4.3-4.7)	4.6 (4.5-4.7)	4.5 (4.3-4.7)	0.14
Bilirubin (mg/dL)	0.9 (0.7-1.1)	0.8 (0.7-1.1)	0.9 (0.7-1.1)	0.30
AST (U/L)	40 (29-63)	27 (23-33)	47 (33-70)	< 0.00001
ALT (U/L)	61 (36-92)	39 (30-56)	69 (42-104)	0.0002
LDH (U/L)	214 (186-240)	189 (165-231)	219 (191-249)	0.02
ALP (U/L)	254 (214-323)	246 (218-312)	258 (214-323)	0.86
γGT (U/L)	53 (35-82)	44 (26-70)	54 (37-87)	0.21
ChE (U/L)	384 (342-429)	399 (369-445)	376 (340-427)	0.12
Urea nitrogen (mg/dL)	13 (11-15)	13.2 (12-14.5)	13 (11-15)	0.65
Creatinine (mg/dL)	0.70 (0.58-0.82)	0.79 (0.62-0.84)	0.66 (0.57-0.82)	0.17
Uric acid (mg/dL)	5.6 (4.8-6.8)	6.0 (5.2-7.5)	5.6 (4.8-6.6)	0.26
eGFR (mL/min/1.73 m^2^)	79 (69-89)	77 (59-88)	79 (70-89)	0.53
Total cholesterol (mg/dL)	209 (181-233)	218 (175-247)	206 (182-230)	0.41
Triglycerides (mg/dL)	119 (91-156)	122 (88-165)	119 (93-156)	0.95
LDL-cholesterol (mg/dL)	134 (109-152)	136 (103-155)	131 (113-147)	0.80
HDL-cholesterol (mg/dL)	52 (45-59)	53 (45-61)	52 (45-57)	0.59
Glucose (mg/dL)	106 (97-120)	99 (92-112)	107 (98-122)	0.02
Insulin (μU/mL)	12 (7.3-17.6)	7.5 (5.1-11.5)	13.1 (8.2-17.9)	0.002
HbA1c (%)	5.8 (5.6-6.4)	5.8 (5.4-6.0)	5.9 (5.6-6.6)	0.02
HOMA-IR	3.2 (1.9-4.8)	2.0 (1.2-3.2)	3.5 (2.2-4.9)	0.001
IgG (mg/dL)	1261 (1089-1535)	1264 (1118-1524)	1261 (1086-1541)	0.77
IgM (mg/dL)	94 (65-126)	83 (54-113)	97 (72-129)	0.18
IgA (mg/dL)	269 (180-333)	252 (185-293)	272 (180-349)	0.56
CRP (mg/dL)	0.10 (0.04-0.17)	0.05 (0.03-0.16)	0.08 (0.05-0.17)	0.13
Hyaluronic acid (mg/dL)	40 (24-74)	29 (23-52)	47 (25-88)	0.08
Type 4 collagen 7S (mg/dL)	4.5 (3.7-5.5)	3.7 (3.6-4.5)	4.5 (3.8-6.3)	0.003
CK18 fragment (U/L)	298 (155-561)	164 (121-293)	351 (178-645)^1^	0.0003
Histological findings				
Steatosis 1/2/3	43/57/26	15/9/2	28/48/24	0.004
Ballooning 0/1/2	26/68/32	26/0/0	0/68/32	< 0.00001
Lobular inflammation 0/1/2/3	7/61/53/5	3/19/4/0	4/42/49/5	0.0003
Fibrosis 0/1/2/3/4	27/56/14/22/7	16/10/0/0/0	11/46/14/22/7	< 0.00001

1 *n* = 96. Data are expressed as number (percentage) or median (25^th^, 75^th^ percentiles). Histological findings were scored according to the criteria proposed by Kleiner et al[22] Comparisons between NAFL and NASH groups were carried out using the χ^2^ test or Mann-Whitney *U* test. BMI: Body mass index; MCV: Mean corpuscular volume; MCH: Mean corpuscular hemoglobin; MCHC: Mean corpuscular hemoglobin concentration; PT-INR: International ratio of prothrombin time; APTT: Activated partial thromboplastin test; FIBG: Fibrinogen; AST: Aspartate aminotransferase; NAFL: Nonalcoholic fatty liver; NASH: Nonalcoholic steatohepatitis; ALT: Alanine aminotransferase; LDH: Lactate dehydrogenase; eGFR: Estimated glomerular filtration rate; ALP: Alkaline phosphatase; γGT: γ-glutamyltransferase; ChE: Cholinesterase; LDL: Low-density lipoprotein; HDL: High-density lipoprotein; HbA1c: Hemoglobin A1c; HOMA-IR: Homeostasis model assessment for insulin resistance; CRP: C-reactive protein; CK18: Cytokeratin 18.

### Relationships between LBP/EndoCab IgG levels and histological findings

**Figure 2:**
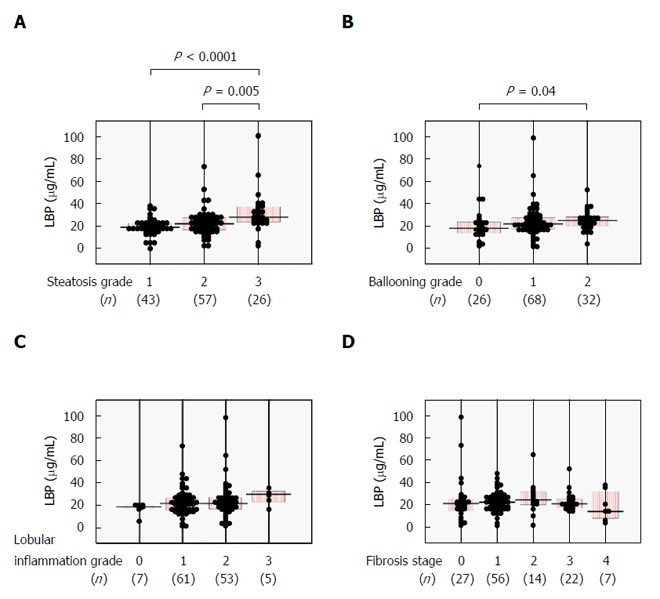
Relationships between serum lipopolysaccharide-binding protein and histological findings in 126 biopsy-proven nonalcoholic fatty liver disease patients. A: Steatosis (1-3); B: Ballooning (0-2); C: Lobular inflammation (0-3); and D: Fibrosis (0-4) scores. Comparisons among subgroups were conducted using one-way ANOVA with Bonferroni’s correction. LBP: Lipopolysaccharide-binding protein.

We next examined whether serum LBP levels correlated with histological severity in NAFLD patients. Serum LBP was significantly higher in the steatosis grade 3 group as compared with the steatosis grade 1 or 2 groups (Figure 2A) and was positively correlated with steatosis score (Table 2). Significantly increased serum LBP was seen in the ballooning grade 2 group over the ballooning grade 0 group (Figure 2B), with a positive correlation between LBP and ballooning score (Table 2). There were no significant relationships between serum LBP and lobular inflammation grade (Figure 2C and Table 2) or fibrosis stage (Figure 2D and Table 2).

**Table 2:** Correlations between lipopolysaccharide-binding protein/EndoCab IgG and individual pathological features

	**LBP**		**EndoCab IgG**	
	***r***	***P* value**	***r***	***P* value**
Steatosis	0.38	< 0.0001	-0.10	0.25
Ballooning	0.23	0.01	0.11	0.22
Lobular inflammation	0.13	0.14	0.05	0.94
Fibrosis	0.03	0.74	0.13	0.13

Histological findings were scored according to the criteria proposed by Kleiner et al[22] Correlation coefficients (*r*) and *P* values were calculated by Spearman’s test. LBP: Lipopolysaccharide-binding protein.

**Figure 3:**
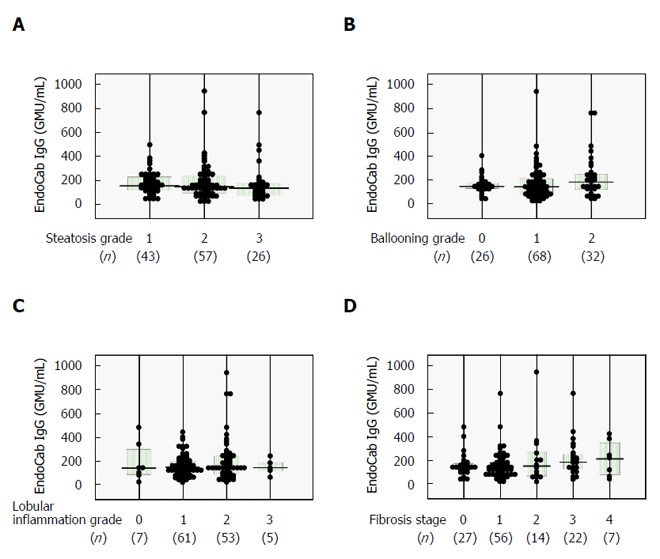
Relationships between serum EndoCab IgG and histological findings in 126 biopsy-proven nonalcoholic fatty liver disease patients. A: Steatosis (1-3); B: Ballooning (0-2); C: Lobular inflammation (0-3); and D: Fibrosis (0-4) scores. Comparisons among subgroups were conducted using one-way ANOVA with Bonferroni’s correction.

**Figure 4:**
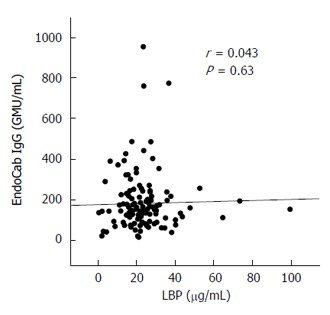
Correlation between serum lipopolysaccharide-binding protein and EndoCab IgG in 126 biopsy-proven nonalcoholic fatty liver disease patients. A correlation efficient (*r*) and *P* value were calculated using Spearman’s test. LBP: Lipopolysaccharide-binding protein.

Similar analyses were carried out for serum EndoCab IgG, which yielded comparable findings among all subgroups (Figure 3) and no correlations with histological scores (Table 2). The lack of agreement between LBP and EndoCab IgG (Figure 4) may explain the discrepant results between LBP and EndoCab IgG for histological scores.

### Correlations between LBP/EndoCab levels and NAS

**Figure 5:**
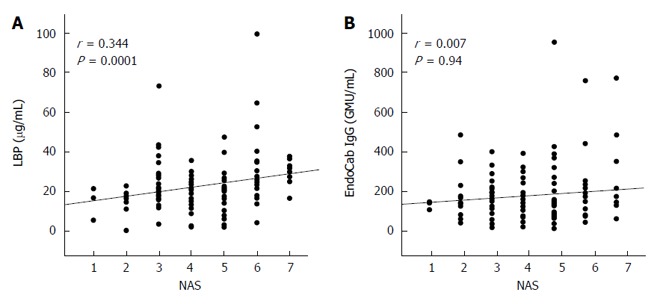
Correlation of nonalcoholic fatty liver disease activity score with serum lipopolysaccharide-binding protein (A) and EndoCab IgG (B) in 126 biopsy-proven nonalcoholic fatty liver disease patients. Correlation efficients (*r*) and *P* values were calculated using Spearman’s test. LBP: Lipopolysaccharide-binding protein; NAS: Nonalcoholic fatty liver disease activity score.

There was a significant positive correlation for serum LBP and NAS, an indicator of histological NAFLD activity, but none for EndoCab IgG (Figure 5).

### Correlations between LBP/EndoCab levels and laboratory data

Serum LBP was significantly positively correlated with AST, ALT, leukocyte count, fibrinogen (FIBG), C-reactive protein (CRP), and CK18 fragment (Table 3), while EndoCab IgG was correlated positively with age, HbA1c, IgG, IgA, and hyaluronic acid and negatively with platelet count (Table 3). Factor analysis using several laboratory data parameters distinguished a clear separation between the LBP and EndoCab IgG groups; the former group included inflammation-related parameters, such as CRP, FIBG, and leukocyte count, whereas the latter group contained HbA1c, age, and hyaluronic acid. Multivariate linear regression analysis revealed that steatosis score (*P* = 0.0033), circulating CRP (*P* = 0.0032), AST (*P* = 0.0151), and FIBG (*P* = 0.0181) were independent predictors of LBP.

**Table 3:** Correlations between lipopolysaccharide-binding protein/EndoCab IgG and routine clinical data

	**LBP**		**EndoCab IgG**	
	***r***	***P* value**	***r***	***P* value**
Age	-0.10	0.25	0.34^1^	0.0001^1^
BMI	0.11	0.23	-0.02	0.86
Leukocyte	0.31^1^	0.0005^1^	-0.07	0.44
Erythrocyte	0.11	0.23	-0.23	0.01
Hemoglobin	0.02	0.87	-0.20	0.024
Hematocrit	0.05	0.55	-0.22	0.013
MCV	-0.15	0.10	0.08	0.37
MCH	-0.15	0.92	0.10	0.25
MCHC	-0.15	0.11	0.02	0.81
Platelet	0.22	0.015	-0.29	0.001
PT-INR	-0.13	0.15	0.12	0.18
APTT	0.06	0.51	0.08	0.37
FIBG	0.30^1^	0.0009^1^	-0.05	0.59
Total protein	-0.01	0.91	0.12	0.18
Albumin	-0.02	0.84	-0.17	0.06
Bilirubin	-0.23	0.01	-0.03	0.73
AST	0.38^1^	< 0.0001^1^	0.07	0.44
ALT	0.35^1^	< 0.0001^1^	-0.09	0.31
LDH	0.24	0.006	0.13	0.16
ALP	0.16	0.07	0.08	0.39
γGT	0.21	0.02	-0.06	0.53
ChE	-0.04	0.69	-0.21	0.02
Urea nitrogen	-0.24	0.007	-0.08	0.35
Creatinine	-0.05	0.60	-0.26	0.004
Uric acid	0.07	0.47	-0.21	0.02
eGFR	0.15	0.12	-0.02	0.85
Total cholesterol	0.09	0.31	0.02	0.86
Triglycerides	0.07	0.46	0.11	0.22
LDL-cholesterol	0.08	0.37	0.001	0.99
HDL-cholesterol	-0.06	0.54	-0.001	0.99
Glucose	-0.08	0.35	0.19	0.04
Insulin	0.17	0.07	0.01	0.96
HbA1c	0.05	0.59	0.27	0.002
HOMA-IR	0.14	0.14	0.04	0.65
IgG	-0.09	0.33	0.30^1^	0.0009^1^
IgM	0.12	0.19	-0.02	0.80
IgA	-0.10	0.28	0.25	0.006
CRP	0.47^1^	< 0.0001^1^	0.05	0.61
Hyaluronic acid	-0.08	0.42	0.36^1^	0.0002^1^
Type 4 collagen 7S	-0.04	0.65	0.17	0.08
CK18 fragment	0.28	0.002	0.002	0.98

1 Items with *r* ≥ 0.3 and *P* < 0.05. Correlation coefficients (*r*) and *P* values were calculated by Spearman’s test. BMI: Body mass index; MCV: Mean corpuscular volume; MCH: Mean corpuscular hemoglobin; MCHC: Mean corpuscular hemoglobin concentration; PT-INR: International ratio of prothrombin time; APTT: Activated partial thromboplastin test; FIBG: Fibrinogen; AST: Aspartate aminotransferase; ALT: Alanine aminotransferase; LDH: Lactate dehydrogenase; eGFR: Estimated glomerular filtration rate; ALP: Alkaline phosphatase; γGT: γ-glutamyltransferase; ChE: Cholinesterase; LDL: Low-density lipoprotein; HDL: High-density lipoprotein; HbA1c: Hemoglobin A1c; HOMA-IR: Homeostasis model assessment for insulin resistance; CRP: C-reactive protein; CK18: Cytokeratin 18.

## DISCUSSION

To our knowledge, this is the first study examining the relationship between two surrogate LPS markers and histological severity in NAFLD. Unexpectedly, serum EndoCab IgG did not correlate with any histological findings, nor was serum LBP associated with lobular inflammation grade or fibrosis stage. Based on these results, we could not conclude whether LPS played a crucial role in hepatitis development or fibrosis progression in NAFLD patients, which deviated from findings obtained in murine models.

Although serum LPS is reportedly elevated in NASH patients[23], the direct measurement of LPS has several flaws. First, LPS has a very short half-life and is rapidly eliminated in the liver, and thus serum LPS values rarely reflect actual endotoxemia. Second, the limulus amoebocyte lysate assay is widely used for LPS determination but may be influenced by exogenous LPS contamination due to its high sensitivity. The assay is also disrupted by detergents, urea, and pH[24]. We therefore adopted the more stable biomarkers LBP and EndoCab IgG as indicators of endotoxemia.

Since LBP is rapidly induced by LPS and EndoCab IgG reflects the immune response to endotoxin core, these biomarkers measure acute/intermittent and chronic/persistent endotoxemia, respectively. Previous studies have examined either serum LBP or EndoCab IgG in NAFLD patients. LBP was similar[25] between control and NAFLD subjects and increased in NAFLD/NASH patients with severe fibrosis[26,27]. EndoCab IgG was comparable[28] between control and NAFLD/NASH groups and higher in NASH[29]. However, the number of NAFLD patients was relatively small (less than 40) in these investigations. Moreover, there have been no reports simultaneously measuring LBP and EndoCab IgG in histologically-proven NAFLD patients or examining these factors for associations with histological findings. The absence of a correlation between LBP and EndoCab IgG has been supported by a large recent study of 920 participants[14]. Although LBP and EndoCab IgG were simultaneously measured in the previous study[14], histological evaluation of the liver was not performed.

In the present series, the severity of lobular inflammation and fibrosis did not correlate with either serum LBP or EndoCab IgG. EndoCab IgG tended to associate with age, hyaluronic acid, and IgG, which was indicative of the possibility of nonspecific IgG elevation due to chronic liver disease and/or aging. Thus, evidence that LPS promoted fibrosis and hepatitis in the context of conventional human NAFLD/NASH could not be demonstrated.

The degree of steatosis correlated with serum LBP level. An earlier study also demonstrated a significant correlation for serum LBP and intrahepatic triglyceride content as determined by proton-magnetic resonance spectroscopy (*r* = 0.366, *P* < 0.001)[14], but not for EndoCab IgG. These similar findings prompted us to consider that LBP might be induced independently of LPS in NAFLD patients. Indeed, the fact that disruption of the LBP-encoding gene in mice decreased basal expression levels of fatty acid-synthesizing enzymes and suppressed steatogenesis[30] implied a direct link between increased LBP and hepatosteatosis. LBP is also up-regulated in hypertrophied adipocytes and acts as an adipokine[31]. Along with CRP and FIBG, LBP is an acute phase reactant as well. It is noteworthy that LBP can be induced by interleukin-6 (IL-6)[32], a pro-inflammatory cytokine, in hepatocytes. IL-6 is markedly up-regulated in steatotic livers[33], indicating a possible link among steatosis, IL-6, and LBP. Although the possibility that LPS directly induced steatosis cannot be ruled out completely, the above findings corroborate a possible association between serum LBP and steatosis independently of LPS/endotoxemia.

This study uncovered a weak, but statistically significant, correlation between serum LBP level and the incidence of ballooned hepatocytes. In hepatocytes with ballooning degeneration, activated c-Jun N-terminal kinase (JNK) and ensuing lipoapoptosis have been documented[34]. *Escherichia coli* LPS induces LBP in human oral keratinocytes through the activation of JNK in addition to nuclear factor kappa B and p38 mitogen-activated protein kinase[35]. Therefore, a positive relationship between LBP expression and ballooning score might reflect activated JNK-mediated signaling in degenerated hepatocytes.

Yuan et al[36] demonstrated that circulating LPS levels in pediatric NASH patients were distributed dichotomously at either high or normal levels, suggesting that endotoxemia was present in specific NASH patients only. Serum LPS levels exhibited no impact on NAS or fibrosis stage and no meaningful relationship was detected between LPS and the proportion of intestinal Gram-negative bacteria. Therefore, they concluded that gut microbiome composition did not contribute to endotoxemia in NASH, nor was endotoxemia always required in the pathogenesis of NASH, which were partially consistent with our results. The observation that antibiotics/probiotics can attenuate NAFLD may not directly support a key role of LPS in NASH development since intestine-derived metabolites/toxicants other than LPS, such as deoxycholic acid and ceramides, promote NAFLD/NASH development[37,38]. The therapeutic use of probiotics/prebiotics has not been supported by high-quality clinical studies[39]. Additionally, the notion that increased hepatic TLR4 expression in NASH indicates an important role of portal endotoxemia may be inappropriate since TLRs are activated by several molecules other than LPS, including palmitic acid[40].

A key limitation of this study was that it did not directly measure LPS concentrations. Since the present investigation used cryogenically stored samples, we were concerned about the accuracy of LPS value measurement due to the abovementioned flaws in the LPS assay system. Future studies will benefit from assessment of LPS using freshly prepared serum samples along with improvements in LPS assay systems. Another limitation was that we could not examine the association between LBP and *PNPLA3* polymorphisms, which might impact the degree of hepatic steatosis. However, a recent study showed no relationship between *PNPLA3* variants and circulating LBP levels in chronic hepatitis C patients[41].

It is reasonable to consider that chronic LPS challenge to steatotic livers, such as NAFLD accompanied with severe gingivitis or inflammatory bowel disease, may be detrimental to liver condition. However, establishing a key contribution of LPS to the histological severity of human primary NASH was not possible in the current study. The lack of appropriate endotoxin markers is a major limitation at present, as is the low sensitivity/specificity of LBP/EndoCab IgG assays. Further improvements in LPS detection systems may provide novel information on the role of LPS/endotoxemia in the pathogenesis of conventional NASH.

## ACKNOWLEDGMENTS

The authors thank Mr. Trevor Ralph for his editorial assistance.

## COMMENTS

### Background

Recent murine studies have demonstrated a key role of endotoxin/lipopolysaccharide (LPS) in the onset of nonalcoholic steatohepatitis (NASH). The contribution of intestinal bacterial overgrowth, increased intestinal permeability, and portal endotoxemia to the progression from nonalcoholic fatty liver (NAFL) to NASH has attracted considerable recent attention. However, evaluating the LPS contribution to human NASH is challenging since LPS is rapidly eliminated in the liver, and therefore venous LPS concentrations often do not reflect portal ones.

### Research frontiers

Circulating LPS-binding protein (LBP) and the anti-endotoxin core immunoglobulin G (EndoCab IgG) antibody are commercially available surrogate markers of endotoxemia that are more stable than LPS. The research hotspot is to examine whether these endotoxemia markers correlate with histological severity in nonalcoholic fatty liver disease (NAFLD).

### Innovations and breakthroughs

This is the first study simultaneously measuring two surrogate endotoxemia markers, LBP and EndoCab IgG, in biopsy-proven NAFLD patients in order to assess for relationships with the histological scores of NAFLD. Serum LBP concentration was significantly increased in NASH patients and was correlated with steatosis and ballooning scores, but not with the severity of lobular inflammation or fibrosis. Serum EndoCab IgG concentration was comparable between NASH and NAFL patients.

### Applications

It is reasonable to consider that chronic LPS challenge to steatotic livers, such as NAFLD accompanied with severe gingivitis or inflammatory bowel disease, may be detrimental to liver condition. However, the contribution of LPS to the histological severity of human primary NASH could not be confirmed in the current study. The lack of appropriate endotoxin markers is a major limitation at present, as is the low sensitivity/specificity of LBP/EndoCab IgG assays. Further improvements in LPS detection systems may provide novel information on the role of LPS/endotoxemia in the pathogenesis of conventional NASH.

### Terminology

NAFLD is a chronic liver disease increasing worldwide that includes a wide spectrum of disorders, ranging from NAFL to NASH and resultant liver cirrhosis and hepatocellular carcinoma. NASH is characterized by the presence of hepatocyte ballooning, lobular inflammation and/or various degrees of fibrosis in addition to macrovesicular steatosis. Increased intestinal permeability and ensuing portal endotoxemia is presumed to be one of the contributors to the progression of NASH.

### Peer-review

This report, written by Kitabatake et al, is of an important retrospective study that has been carried out to disclose pathological mechanisms of NAFLD/NASH as well as its diagnosis. Especially, the close relationship between LBP and NASH is very interesting.
